# Lower-limb strength and power characteristics in relation to 180° change of direction ability in elite female basketball players

**DOI:** 10.3389/fspor.2025.1732018

**Published:** 2026-01-13

**Authors:** Hiroki Ogata, Daichi Yamashita, Naoto Nishikawa, Toshiharu Yokozawa, Masako Hoshikawa

**Affiliations:** 1 Graduate School of Physical Education, National Institute of Fitness and Sports in Kanoya, Kagoshima, Japan; 2 Japan Basketball Association, Tokyo, Japan; 3 Japan Institute of Sports Sciences, Japan High Performance Sports Center, Tokyo, Japan

**Keywords:** lateral shuffle, 180° turn, ground reaction force, isometric mid-thigh pull, countermovement jump

## Abstract

This study examined the relationships among lower-limb strength, power, and horizontal ground reaction force (GRF) during change of direction (COD) tasks in elite female basketball players. Sixteen athletes completed an isometric mid-thigh pull (IMTP), a countermovement jump (CMJ), a lateral shuffle, and 180° turns. For the COD task, the turning distance was individualized based on each participant's standing height. Dual portable uniaxial force-plates were used to measure IMTP peak force and the rate of force development (RFD) over 0–200 ms (RFD_200_) and 0–250 ms (RFD_250_). CMJ jump height and phase-specific peak power (braking and propulsive) were also quantified, with all variables normalized to body mass. Horizontal GRFs during plant-foot contact in the COD tasks were recorded using triaxial force-plates, and both peak and mean GRF relative to body mass were analyzed. Mean horizontal GRF during both the lateral shuffle and the 180° turn showed consistent, significant positive correlations with IMTP peak force (*r* = 0.56–0.80, all *p* < 0.05). In contrast, correlations between mean horizontal GRF and IMTP RFD were limited, reaching significance only during the right lateral shuffle (RFD_250_: *r* = 0.51, *p* = 0.04) and the left 180° turn (RFD_200_ and RFD_250_: both *r* = 0.68, *p* < 0.01). Peak horizontal GRF showed negligible associations with most IMTP or CMJ variables, except for IMTP peak force during the left 180° turn (*r* = 0.51, *p* = 0.045). Associations with CMJ metrics were modest; jump height correlated significantly with mean horizontal GRF only during the right lateral shuffle (*r* = 0.50, *p* = 0.047), whereas CMJ peak braking and propulsive power showed no significant relationships. These findings emphasize the importance of maximal isometric strength for COD performance in this population and highlight the need to select assessment indices aligned with the task-specific demands of strength and conditioning programs.

## 1 Introduction

Basketball is an intermittent sport characterized by high-intensity movements such as acceleration, deceleration, changes of direction (COD), and jumping, all of which are major determinants of basketball performance (1, 2). Among these, COD maneuvers are repeatedly executed during games to switch between offense and defense and to create space, making them a key component of competitive performance (3, 4). Particularly in women's basketball, research interest has been growing, with recent reviews highlighting that female players are exposed to substantial internal and external training and competition loads (5). Accordingly, identifying the potential neuromuscular factors of COD ability has direct practical value for designing training programs and evaluating athletes' performance.

COD ability has been traditionally evaluated using the total completion time of specific COD tests, which have been associated with lower-limb strength and power (6–11). For example, in female basketball players, greater isometric and eccentric strength has been linked to faster completion times in tests that include a 180° COD component, such as the 505 and T-test (6, 7). However, many COD tests include a substantial proportion of linear sprinting, and total test time correlates strongly with short sprint performance (12–14), which may limit its specificity as a measure of COD performance (12).

From a biomechanical perspective, a COD maneuver typically consists of braking, transition, and propulsion (re-acceleration) phases (6). The final step, commonly referred to as the plant foot, marks the critical point at which these phases occur in rapid succession. This sequence involves distinct modes of muscle contraction, including eccentric strength for braking, isometric strength during the plant phase, and concentric strength during propulsion (6). Efficient transitions from deceleration to propulsion through the plant foot also require effective utilization of the stretch–shortening cycle. In particular, horizontal GRF during plant-foot contact, when normalized to body mass according to Newton's Second Law (***F*** = *m**a***), represents the horizontal acceleration of an athlete's center of mass (COM). This metric provides a more direct indicator of COD ability and has been reported to strongly associate with COD test time (15, 16). Dos'Santos et al. (2020) reported that plant-foot horizontal GRF is a primary determinant of completion time in a 180° turn (505 test) (15). Similarly, Shimokochi et al. (2013) showed that in a lateral shuffle COD task among female basketball players, those with a larger lateral cutting index (defined as the COM velocity at take-off divided by contact time) exhibited greater peak horizontal GRF (17). Nevertheless, studies examining the links between horizontal GRF during COD tasks and lower-limb force production capacity are lacking.

To quantify such lower-limb force-production capacities, portable force-plate (FP) assessments such as the isometric mid-thigh pull (IMTP) and countermovement jump (CMJ) have been widely used for athlete monitoring and performance profiling (18). These tests are considered indicative of partly distinct neuromuscular qualities (19). The IMTP allows for the evaluation of peak vertical ground reaction force (GRF) and rate of force development (RFD) (20, 21), both of which are considered essential for maintaining postural control and producing force in the intended direction during COD movements (7, 22). In addition to overall jump height, subphase analysis of the CMJ enables separate quantification of power during the braking and propulsive phases (23, 24). Such analysis provides a multifaceted understanding of neuromuscular function (19). However, to the best of our knowledge, only Spiteri et al. (2015) reported that faster elite female basketball players in the 180° turn (505 test) possessed higher IMTP peak force and demonstrated greater vertical GRF; however, horizontal GRF was not analyzed (6). Therefore, it is not well established how commonly used FP-derived strength and power metrics relate to the horizontal GRF demands of COD maneuvers in elite female basketball players.

Given that lateral shuffles and 180° turns are common in basketball (3, 7, 17, 25), clarifying how IMTP- and CMJ-derived strength and power metrics relate to plant foot horizontal GRF in these tasks is warranted. Since the IMTP and CMJ are time-efficient and reliable (20, 26–28), establishing this relationship would enhance the ecological validity of these tests, offering direct applications for training design and performance profiling. Therefore, this study aimed to examine the relationships between IMTP- and CMJ-derived strength and power metrics and the horizontal GRF produced during lateral shuffle and 180° turn COD tasks in elite female basketball players. We hypothesized that athletes who exhibit greater peak and mean horizontal GRF at the plant foot would demonstrate higher IMTP peak force and RFD and would produce greater power in both the braking and propulsive phases of the CMJ.

## 2 Methods

### 2.1 Participants

Sixteen elite female basketball players (age: 23.9 ± 4.4 years; height: 173.1 ± 7.9 cm; body mass: 67.6 ± 10.4 kg) from the Japan Women's National Team training camp participated in this study. At the time of testing, all the athletes were free from restrictions on sport participation. According to McKay et al. (2022) athlete classification, eight players were classified as Tier 5 (world-class) and eight as Tier 4 (elite/international) (29).

The study was approved by the Institutional Ethics Committee of the Japan Institute of Sports Sciences (no. 2021-057-3). All tests were conducted at the beginning of a training session during the national training camps as part of the team assessment. Prior to testing, all participants were informed of the potential benefits and risks of the test, and written consent was obtained regarding the potential use of their data for research purposes. Information on the study's purpose and the option to opt out were made publicly available on the Japan High Performance Sports Center website (https://www.jpnsport.go.jp/hpsc/business/ourwork/tabid/1322/Default.aspx), allowing athletes to opt out without facing any disadvantages.

### 2.2 Procedures

All measurements were conducted in a controlled indoor training facility. After completing a standardized dynamic warm-up supervised by a strength and conditioning coach, each participant performed familiarization trials at 50%, 70%, and 90% of perceived maximal effort for every test task. The order of the tests (IMTP, CMJ, and COD tasks) was randomized for each participant. Sufficient rest was provided between tests, and the next test commenced only after the absence of visible signs of fatigue had been confirmed.

A portable dual-uniaxial FP system (1,000 Hz; Hawkin Dynamics Inc., G3 Force Plates, Maine, USA) was used to quantify kinetic variables for both CMJ and IMTP. The system hardware and software had previously demonstrated adequate validity (30, 31). Standardized verbal cues and encouragement were provided by the researchers during each attempt.

The IMTP was performed on the FPs using a portable isometric pull rack with adjustable pinholes (Hawkin Dynamics Inc., Maine, USA), following the recommendations of Comfort et al. (2019) (20). Body weight was first recorded using the system's “weigh-in” mode while participants stood still on the FPs; the lowest 1-s average of the vertical GRF was used (32). Body mass was then calculated by dividing body weight by gravitational acceleration (9.81 m·s^−2^). The IMTP posture replicated the second-pull position of the clean: athletes grasped the bar using lifting straps with feet approximately shoulder-width apart, a knee angle set between 125° and 145°, and a hip angle between 140° and 150° (20). Participants were instructed to “push your feet into the ground as fast and as hard as possible (20).” After holding a brief still starting position, each trial began on the verbal countdown “3, 2, 1, GO!” and continued for at least 3 s (20). Strong verbal encouragement was given throughout each trial. A maximum of two trials were conducted. Trials were repeated if baseline force fluctuations exceeded 50 N, if a clear countermovement occurred, or if the peak force appeared at the end of the trial (20, 33).

For the CMJ, participants performed three trials on the FPs with their hands on their hips. They were instructed to execute rapid countermovement to a knee angle of approximately 90°, followed by an immediate maximal vertical jump. Trials that deviated from the criteria were repeated at the examiner's discretion. Thirty to sixty seconds of rest were provided between trials. For safety, the area around the FPs was surrounded by a custom-built lightweight foam barrier (Hard Foam Surround, 50″ × 46″; Hawkin Dynamics, Maine, USA).

Two COD tasks were administered: the 180° turn and the lateral shuffle. In both tasks, the outcome variable was the horizontal GRF (relative to body mass) during plant-foot contact. Measurements were obtained using four in-ground consecutive FPs (1,000 Hz; 0.9 m × 0.6 m; Type 9281EA, Kistler, Winterthur, Switzerland), from which GRF data during plant-foot contact were recorded. For each task, two consecutive trials per plant foot (left and right) were administered in self-selected order, resulting in eight total trials per athlete. The start and direction-change points were marked on the plates, and the distance between them was adjusted based on each participant's height (17). All participants were instructed to perform “as fast as possible” and “at maximal effort.” Each trial was initiated by a verbal cue once participants had maintained their starting stance for at least 1 s to enable body-weight calibration. Trials were repeated if a participant slipped, turned before crossing the designated line, or failed to make full contact with the FPs.

In the lateral-shuffle task, participants began in an athletic stance perpendicular to the starting line, with the lateral border of the plant foot just behind the line. They then performed a two-step lateral shuffle to the designated turn line, executed a cut, and returned to the start and finish lines once both feet had crossed (17). In the 180°-turn task, participants began in a staggered stance behind the start line, sprinted forward in two steps to the turn line, planted the second step beyond the turn line to execute the 180° turn, and sprinted 5 m to the finish line.

### 2.3 Data analysis

Force and power variables from the IMTP and CMJ were exported as CSV files from the Hawkin Dynamics cloud-based software and analyzed using Microsoft Excel (Microsoft Corporation, Redmond, WA, USA). For the CMJ, jump height and peak power during the braking and propulsive phases were extracted, with peak power values normalized to body mass. The mean value across the three trials was used for further analysis.

For the IMTP, peak force and RFD over 0–200 ms (RFD_200_) and 0–250 ms (RFD_250_) were calculated (8, 34). All IMTP variables were normalized to body mass, and the mean values across trials were used for statistical analysis.

For the COD tasks, peak and mean horizontal GRFs during plant-foot contact, defined by a vertical GRF greater than 20 N (35) were calculated and normalized to body mass. Body weight was calculated using a 0.5-s (500-data-point) moving average of vertical GRF with the smallest standard deviation (SD) during quiet standing (36). A one-frame sliding window was applied across the quiet-standing phase, and the body-weight value from the window with the smallest SD was adopted for analysis. We confirmed that the coefficient of variation of vertical GRF within the selected window for each trial was less than 1.5%. GRF signals were smoothed using a fourth-order low-pass Butterworth filter with a cut-off frequency of 25 Hz (15). For statistical analyses, the peak and mean horizontal GRF values during plant-foot contact were averaged across the two trials.

### 2.4 Statistical analysis

Statistical analyses were performed using IBM SPSS Statistics (version 29, IBM Inc., Armonk, NY, USA), with the significance level set at *p* < 0.05. The normality of each variable was assessed using the Shapiro–Wilk test and Q–Q plots. All variables were deemed approximately normally distributed, as indicated by the Shapiro–Wilk test (*p* ≥ 0.05) and/or the Q–Q plot exhibiting an approximately linear pattern with only minor deviations. Accordingly, all data are presented as mean ± SD.

Intrasession reliability across *k* trials was evaluated using the intraclass correlation coefficient (ICC_2,k_). The ICC_2,k_ values were interpreted according to the following thresholds: 0.1 = low, 0.3 = moderate, 0.5 = high, 0.7 = very high, 0.9 = nearly perfect, and 1.0 = perfect (37).

Pearson's product-moment correlation coefficients were calculated to assess the associations between horizontal GRF and IMTP and CMJ variables. Correlation magnitudes were interpreted as follows: 0.10–0.29 = small, 0.30–0.49 = moderate, 0.50–0.69 = large, 0.70–0.89 = very large, 0.90–0.99 = almost perfect, and 1.0 = perfect (38). Statistical signiﬁcance was set at *p* < 0.05.

## 3 Results

Descriptive statistics and within-session reliability for all measurements are presented in Table 1. All variables demonstrated acceptable reliability, with ICC_2,k_ values exceeding 0.70 (range: 0.72–0.98), indicating very high to nearly perfect reliability.

**Table 1:** Descriptive statistics for performance variables.

Test	Foot	Variable	Mean ± SD	ICC (2,k) (95%CI LL, UL)
Isometric mid-thigh pull		Peak force (N/kg)	37.62 ± 3.56	0.97 (0.90, 0.99)
		RFD 0–200 ms (N/s/kg)	100.36 ± 17.26	0.82 (0.47, 0.94)
		RFD 0–250 ms (N/s/kg)	89.15 ± 13.48	0.80 (0.42, 0.93)
Countermovement jump		Jump height (m)	0.285 ± 0.04	0.98 (0.95, 0.99)
		Peak braking power (W/kg)	−19.76 ± 3.30	0.90 (0.76, 0.96)
		Peak propulsive power (W/kg)	43.82 ± 4.94	0.98 (0.95, 0.99)
Lateral shuffle	R	Peak horizontal force (N/kg)	17.89 ± 2.30	0.72 (0.17, 0.90)
	L	Peak horizontal force (N/kg)	18.91 ± 2.94	0.76 (0.30, 0.91)
	R	Mean horizontal force (N/kg)	10.54 ± 1.20	0.93 (0.81, 0.98)
	L	Mean horizontal force (N/kg)	10.78 ± 1.27	0.89 (0.69, 0.96)
180° turn	R	Peak horizontal force (N/kg)	19.83 ± 2.92	0.85 (0.57, 0.95)
	L	Peak horizontal force (N/kg)	19.71 ± 2.90	0.81 (0.16, 0.94)
	R	Mean horizontal force (N/kg)	10.59 ± 1.34	0.83 (0.53, 0.94)
	L	Mean horizontal force (N/kg)	10.73 ± 1.14	0.88 (0.58, 0.96)

RFD_200_, rate of force development at 0–200 ms; RFD_250_, rate of force development at 0–250 ms.

“R” and “L” indicate the right and left plant foot during cutting, respectively.

The correlations between lower-limb strength, power variables, and horizontal GRF during the COD tasks are summarized in Table 2. In the lateral-shuffle, peak horizontal GRF was not significantly correlated with any IMTP or CMJ variables. In contrast, mean horizontal GRF showed significant positive correlations with the IMTP peak force (right: *r* = 0.62, *p* = 0.01; left: *r* = 0.68, *p* < 0.01). On the right side, mean horizontal GRF was also correlated with RFD_250_ (*r* = 0.51, *p* = 0.04) and jump height (*r* = 0.50, *p* = 0.047).

**Table 2:** Correlation coefficients (with 95% confidence intervals: lower, upper) between horizontal ground reaction force and lower limb strength and power characteristics.

			IMTP			CMJ		
Horizontal GRF (N/kg)		Foot	Peak force (N/kg)	RFD 0–200 ms (N/s/kg)	RFD 0–250 ms (N/s/kg)	Jump height (m)	Peak braking power (W/kg)	Peak propulsive power (W/kg)
Lateral shuffle	Peak	R	0.41 (−0.10, 0.75)	0.22 (−0.31, 0.64)	0.31 (−0.22, 0.70)	0.32 (−0.20, 0.71)	−0.46 (−0.78, 0.04)	0.19 (−0.34, 0.63)
		L	0.36 (−0.17, 0.72)	0.09 (−0.43, 0.56)	0.18 (−0.34, 0.62)	0.20 (−0.33, 0.63)	−0.42 (−0.76, 0.09)	0.06 (−0.45, 0.54)
	Mean	R	0.62* (0.18, 0.85)	0.46 (−0.04, 0.78)	0.51* (0.02, 0.80)	0.50* (0.01, 0.80)	−0.35 (−0.72, 0.17)	0.35 (−0.17, 0.72)
		L	0.68** (0.28, 0.88)	0.37 (−0.15, 0.73)	0.44 (−0.07, 0.77)	0.33 (−0.20, 0.71)	−0.30 (−0.69, 0.23)	0.17 (−0.36, 0.61)
180° turn	Peak	R	0.18 (−0.35, 0.62)	0.21 (−0.32, 0.64)	0.19 (−0.34, 0.63)	0.25 (−0.28, 0.66)	−0.37 (−0.73, 0.15)	0.12 (−0.40, 0.58)
		L	0.51* (0.01, 0.80)	0.28 (−0.25, 0.68)	0.35 (−0.18, 0.72)	0.35 (−0.18, 0.72)	−0.49 (−0.79, 0.01)	0.23 (−0.30, 0.65)
	Mean	R	0.56* (0.09, 0.83)	0.44 (−0.07, 0.77)	0.45 (−0.06, 0.77)	0.45 (−0.07, 0.77)	−0.38 (−0.74, 0.14)	0.27 (−0.26,0.68)
		L	0.80** (0.51, 0.93)	0.68** (0.27, 0.88)	0.68** (0.28, 0.88)	0.49 (−0.01, 0.79)	−0.34 (−0.71, 0.19)	0.33 (−0.20, 0.71)

GRF, ground reaction force; RFD200, rate of force development at 0–200 ms; RFD250, rate of force development at 0–250 ms.

“R” and “L” indicate the right and left plant foot during cutting, respectively.

* *p* < 0.05.

** *p* < 0.01.

For the 180°-turn task, peak horizontal GRF was significantly associated with IMTP peak force on the left side (*r* = 0.51, *p* = 0.045), with no other significant associations observed (*r* = −0.49 to 0.35, *p* > 0.05). Mean horizontal GRF correlated positively with IMTP peak force (right: *r* = 0.56, *p* = 0.02; left: *r* = 0.80, *p* < 0.01) and, on the left side, with RFD_200_ and RFD_250_ (both *r* = 0.68, *p* < 0.01). No additional significant relationships were observed.

## 4 Discussion

This study investigated the relationships among lower-limb force and power test performances and horizontal GRF at the plant foot during COD tasks in elite female basketball players. Mean horizontal GRF showed significant positive correlations with IMTP peak force during both right and left lateral shuffles and 180°-turns, whereas its associations with IMTP RFD or CMJ variables were limited. In addition, peak horizontal GRF exhibited few significant correlations with IMTP peak force, RFD, or CMJ variables. These findings suggest that maximal isometric strength, rather than RFD or CMJ-derived power, may represent an important physical characteristic associated with horizontal force production at the plant foot during the COD tasks in this cohort. Additionally, lateral shuffling and 180° turn are embedded in widely used COD tests such as the lane agility test, 505 test, and T-test. This overlap supports the external validity and practical relevance of the COD maneuvers.

Few studies have examined GRF profiles during 180° COD tasks in elite female athletes. In the lateral-shuffle, the peak horizontal GRF observed in this study was comparable to that previously reported for Japanese female collegiate basketball players (18.6 N·kg^−1^) (17). This similarity is notable given that the athletes in the present sample were taller (+7 cm) and heavier (+9 kg) than those in the collegiate cohort. By contrast, the peak (17–20 N·kg^−1^) and mean (∼11 N·kg^−1^) horizontal GRFs recorded during the 180° turn in this study exceeded those reported for male athletes (peak ∼11 N·kg^−1^; mean ∼9 N·kg^−1^) (15, 37). Furthermore, CMJ height and IMTP peak force were similar to or greater than values reported for National Collegiate Athletic Association (NCAA) Division I women's basketball players (8, 39). Collectively, these findings from this group of elite female athletes help to clarify the physical qualities underlying COD performance and provide valuable insights for the design of resistance training programs.

The magnitudes of association between force–power characteristics and horizontal GRF differed between peak and mean GRF values. Many previous studies have demonstrated associations between COD test time and lower-limb strength and power. A recent meta-analysis reported significant associations between COD test time and maximal lower body strength, knee extension strength, reactive strength, and lower body power (40). However, the present results suggest a more specific relationship limited to particular strength qualities. This discrepancy likely reflects differences in how COD performance is defined. Conventional research relies on total COD task time (e.g., the 505 test), which includes substantial linear sprinting components. Consequently, total COD time is strongly influenced by sprint ability and may not isolate COD-specific performance (12, 14, 41). In contrast, the present study defined COD performance based on the horizontal GRF at the plant foot, representing COM acceleration, to more directly capture the physical qualities linked to the COD maneuver itself.

Although IMTP peak force was positively correlated with mean horizontal GRF in both the lateral shuffle and the 180° turn tasks, associations with peak horizontal GRF were largely absent. This difference likely reflects distinct mechanical demands captured by each metric. Previous research has indicated that 180°-turn performance (task time) relates more strongly to mean horizontal GRF than to peak horizontal GRF (15, 37). Peak horizontal GRF typically occurs within the first 10% of contact when the hip, knee, and ankle are still flexing (35), suggesting a predominantly eccentric loading demand. In contrast, mean horizontal GRF at the plant foot reflects both the magnitudes of braking, redirection, and re-acceleration forces, as well as the duration of contact. Shorter contact times are also associated with faster COD performance (15, 37). Therefore, mean horizontal GRF appears to provide a more comprehensive indicator of overall force-production characteristics at the plant foot during COD, compared to peak horizontal GRF.

In the 180° COD task, mean horizontal GRF correlated consistently with IMTP peak force, whereas correlations with IMTP RFD were only moderate to large and statistically significant under limited conditions. These associations were not uniform across tasks and directions: in the lateral shuffle, significant correlations between mean horizontal GRF and IMTP peak force were observed for both right and left sides, whereas in the 180° turn, the corresponding relationships ranged from moderate to very large and were not mirrored in peak horizontal GRF. Previous studies have shown associations between the 180° turn test time and IMTP peak force (6, 10, 11, 34, 42), as well as between maximal back squat strength and COD performance in various female athlete populations (7, 42–44). Meta-analytic evidence also highlights knee extension strength as a primary determinant of COD test time (40). The standard IMTP posture (knee angle 135–145°) produces greater knee extension torque and higher peak force than other joint angles (45), supporting IMTP peak force as a valid index of strength qualities relevant to COD ability. Moreover, plant-foot contact time in 180°-turns typically exceeds 250 ms (46); therefore, short-window RFD indices (RFD_200_ and RFD_250_) may not adequately represent the neuromuscular demands of the task. Taken together, these findings suggest that maximal isometric force capacity may represent an important neuromuscular characteristic contributing to deceleration and re-acceleration during sharp-angle COD maneuvers, but they also indicate that COD performance is likely multifactorial and influenced by additional factors such as inter-limb asymmetries and movement strategy.

In the present study, CMJ metrics were generally not associated with horizontal GRF during COD tasks. For most variables, correlation coefficients were small to moderate and did not reach statistical significance. On the surface, this appears inconsistent with meta-analytic evidence linking COD time with CMJ height and lower-limb power (40). One possible explanation is that the association between CMJ height and COD time diminishes as sprint distance shortens. Falch et al. reported a correlation for a 10-m shuttle but not with a 5-m shuttle (47). In the present study, the turning distance was scaled to each athlete's body height (mean ∼1.73 m), following a previous protocol (17), which may have attenuated the correlation with CMJ performance. Furthermore, while the CMJ assesses bilateral vertical force production, COD tasks require unilateral horizontal force application (15, 48). In elite male basketball players, horizontal GRF in a unilateral lateral CMJ discriminates between faster and slower lateral shuffle times (49), which hypothetically suggests that a bilateral vertical CMJ may not fully capture the mechanical demands placed on the plant foot during COD, particularly during eccentric braking. It is important to acknowledge that this interpretation is based on extrapolating findings from previous literature and not direct evidence from the present study, as unilateral force measures (e.g., unilateral CMJ) and approach velocity were not tested here. This distinction is offered as a possible explanation and highlights areas for future research. Additionally, among national team female soccer players, no preintervention correlation was observed between CMJ peak power and 180° COD test time; however, after a 20-week training intervention, a significantly negative correlation emerged (50). This pattern suggests that training progression can modify the relationships between strength and power metrics and COD ability. Longitudinal research is therefore warranted to clarify how lower-limb strength and power relate to COD ability over time and under specific training interventions.

This study has some limitations. First, although the cohort was highly specific and elite, the small sample size (*n* = 16) reduces statistical power and limits generalizability. Furthermore, the cross-sectional design prevents establishing causal relationships. Second, although COD relies on unilateral force contribution, we analyzed its relationship with bilateral tests (CMJ, IMTP) and did not measure unilateral qualities or reactive strength. Additionally, approach velocity during COD tasks was not measured. Third, this study focused on conventional IMTP metrics (peak force and RFD) and did not compute detailed force–time characteristics (e.g., time to reach specific relative force thresholds or early-phase impulse), which have been linked to sprint and COD performance (8, 10, 51, 52). Thus, unexamined IMTP force–time indices may show additional or stronger associations with horizontal GRF during COD maneuvers. Nevertheless, the emergence of these relationships within a highly homogeneous and specialized elite cohort provides meaningful insight into the potential neuromuscular characteristics associated with COD performance.

## 5 Conclusion

In elite female basketball players, mean horizontal GRF during lateral shuffles and 180°-turns was significantly associated with IMTP peak force, whereas associations with CMJ jump height and phase-specific power (braking and propulsive) were limited. These findings help clarify the physical qualities assessed by portable FP that underlie COD performance and offer valuable insights for designing effective resistance training programs.

## Data Availability

The raw data supporting the conclusions of this article will be made available by the authors, without undue reservation.

## References

[1] Conte D Favero TG Lupo C Francioni FM Capranica L Tessitore A. Time-motion analysis of Italian elite women’s basketball games: individual and team analyses. J Strength Cond Res. (2015) 29:144–50. 10.1519/JSC.0000000000000633 25051006

[2] Koyama T Rikukawa A Nagano Y Sasaki S Ichikawa H Hirose N. High-acceleration movement, muscle damage, and perceived exertion in basketball games. Int J Sports Physiol Perform. (2022) 17:16–21. 10.1123/ijspp.2020-0963 34186509

[3] Sugiyama T Maeo S Kurihara T Kanehisa H Isaka T. Change of direction speed tests in basketball players: a brief review of test varieties and recent trends. Front Sports Act Living. (2021) 3:645350. 10.3389/fspor.2021.645350 33997779 PMC8117963

[4] Abdelkrim NB Chaouachi A Chamari K Chtara M Castagna C. Positional role and competitive-level differences in elite-level men’s basketball players. J Strength Cond Res. (2010) 24:1346–55. 10.1519/JSC.0b013e3181cf7510 20393355

[5] Reina M García-Rubio J Ibáñez SJ. Training and competition load in female basketball: a systematic review. Int J Environ Res Public Health. (2020) 17:2639. 10.3390/IJERPH17082639 32290569 PMC7215482

[6] Spiteri T Newton RU Binetti M Hart NH Sheppard JM Nimphius S. Mechanical determinants of faster change of direction and agility performance in female basketball athletes. J Strength Cond Res. (2015) 29:2205–14. 10.1519/JSC.0000000000000876 25734779

[7] Spiteri T Nimphius S Hart NH Specos C Sheppard JM Newton RU. Contribution of strength characteristics to change of direction and agility performance in female basketball athletes. J Strength Cond Res. (2014) 28:2415–23. 10.1519/JSC.0000000000000547 24875426

[8] Townsend JR Bender D Vantrease WC Hudy J Huet K Williamson C Isometric midthigh pull performance is associated with athletic performance and sprinting kinetics in division I men and women’s basketball players. J Strength Cond Res. (2019) 33:2665–73. 10.1519/JSC.0000000000002165 28777249

[9] Chang C-C Chiang C Chiu C-Y Chow T-H. The relationship between kinematic and kinetic characteristics of countermovement jump and change of direction in elite female basketball players. In: Lin K-P Magjarević R de Carvalho P, editors. International Conference on Biomedical and Health Informatics 2024. Cham: Springer Nature Switzerland (2025). p. 297–313.

[10] Thomas C Comfort P Chiang C-Y Jones PA. Relationship between isometric mid-thigh pull variables and sprint and change of direction performance in collegiate athletes. J Trainol. (2015) 4:6–10. 10.17338/trainology.4.1_6

[11] Thomas C Comfort P Jones PA Dos’Santos T. A comparison of isometric midthigh-pull strength, vertical jump, sprint speed, and change-of-direction speed in academy netball players. Int J Sports Physiol Perform. (2017) 12:916–21. 10.1123/ijspp.2016-0317 27918677

[12] Sayers MGL. Influence of test distance on change of direction speed test results. J Strength Cond Res. (2015) 29:2412–6. 10.1519/JSC.0000000000001045 26049789

[13] Nimphius S Callaghan SJ Spiteri T Lockie RG. Change of direction deﬁcit: a more isolated measure of change of direction performance than total 505 time. J Strength Cond Res. (2016) 30:3024–32. 10.1519/JSC.0000000000001421 26982972

[14] Nimphius S Callaghan SJ Bezodis NE Lockie RG. Change of direction and agility tests: challenging our current measures of performance. Strength Cond J. (2018) 40:26–38. 10.1519/SSC.0000000000000309

[15] Dos’Santos T McBurnie A Thomas C Comfort P Jones PA. Biomechanical determinants of the modified and traditional 505 change of direction speed test. J Strength Cond Res. (2020) 34:1285–96. 10.1519/JSC.0000000000003439 31868815

[16] Jones PA Thomas C Dos'Santos T McMahon JJ Graham-Smith P. The role of eccentric strength in 180◦ turns in female soccer players. Sports. (2017) 5:42. 10.3390/sports5020042 29910402 PMC5968983

[17] Shimokochi Y Ide D Kokubu M Nakaoji T. Relationships among performance of lateral cutting maneuver from lateral sliding and hip extension and abduction motions, ground reaction force, and body center of mass height. J Strength Cond Res. (2013) 27:1851–60. 10.1519/JSC.0b013e3182764945 23085969

[18] Merrigan JJ Stone JD Hornsby WG Hagen JA. Identifying reliable and relatable force–time metrics in athletes—considerations for the isometric mid-thigh pull and countermovement jump. Sports. (2021) 9:4. 10.3390/SPORTS9010004 PMC7824153 33396304

[19] James LP Talpey SW Young WB Geneau MC Newton RU Gastin PB. Strength classification and diagnosis: not all strength is created equal. Strength Cond J. (2023) 45:333–41. 10.1519/SSC.0000000000000744

[20] Comfort P Dos'Santos T Beckham GK Stone MH Guppy SN Gregory Haff G. Standardization and methodological considerations for the isometric midthigh pull. Strength Cond J. (2019) 41:57–79. 10.1519/SSC.0000000000000433

[21] Brady CJ Harrison AJ Flanagan EP Gregory Haff G Comyns TM. A comparison of the isometric midthigh pull and isometric squat: intraday reliability, usefulness, and the magnitude of difference between tests. Int J Sports Physiol Perform. (2018) 13:844–52. 10.1123/ijspp.2017-0480 29182457

[22] Spiteri T Cochrane JL Hart NH Haff GG Nimphius S. Effect of strength on plant foot kinetics and kinematics during a change of direction task. Eur J Sport Sci. (2013) 13:646–52. 10.1080/17461391.2013.774053 24251742

[23] McMahon JJ Suchomel TJ Lake JP Comfort P. Understanding the key phases of the countermovement jump force-time curve. Strength Cond J. (2018) 40:96–106. 10.1519/SSC.0000000000000375

[24] Harper DJ Cohen DD Carling C Kiely J. Can countermovement jump neuromuscular performance qualities differentiate maximal horizontal deceleration ability in team sport athletes? Sports. (2020) 8:76. 10.3390/sports8060076 32471190 PMC7353628

[25] Taylor JB Wright AA Dischiavi SL Townsend MA Marmon AR. Activity demands during multi-directional team sports: a systematic review. Sports Med. (2017) 47:2533–51. 10.1007/s40279-017-0772-5 28801751

[26] Alba-Jiménez C Moreno-Doutres D Peña J. Trends assessing neuromuscular fatigue in team sports: a narrative review. Sports. (2022) 10:33. 10.3390/sports10030033 35324642 PMC8950744

[27] Grgic J Scapec B Mikulic P Pedisic Z. Test-retest reliability of isometric mid-thigh pull maximum strength assessment: a systematic review. Biol Sport. (2022) 39:407–14. 10.5114/biolsport.2022.106149 35309521 PMC8919882

[28] Claudino JG Cronin J Mezêncio B McMaster DT McGuigan M Tricoli V The countermovement jump to monitor neuromuscular status: a meta-analysis. J Sci Med Sport. (2017) 20:397–402. 10.1016/j.jsams.2016.08.011 27663764

[29] McKay AKA Stellingwerff T Smith ES Martin DT Mujika I Goosey-Tolfrey VL Defining training and performance caliber: a participant classification framework. Int J Sports Physiol Perform. (2022) 17:317–31. 10.1123/ijspp.2021-0451 34965513

[30] Badby AJ Mundy PD Comfort P Lake JP McMahon JJ. The validity of hawkin dynamics wireless dual force plates for measuring countermovement jump and drop jump variables. Sensors. (2023) 23:4820. 10.3390/s23104820 37430733 PMC10224001

[31] Merrigan JJ Stone JD Galster SM Hagen JA. Analyzing force-time curves: comparison of commercially available automated software and custom MATLAB analyses. J Strength Cond Res. (2022) 36:2387–402. 10.1519/JSC.0000000000004275 35916879

[32] Soriano MA Paredes V Comfort P Jiménez-Ormeño E Areces-Corcuera F Giráldez-Costas V “You are not wrong about getting strong:” an insight into the impact of age group and level of competition on strength in Spanish football players. Int J Sports Physiol Perform. (2024) 19:629–36. 10.1123/ijspp.2023-0510 38648884

[33] Dos'Santos T Jones PA Comfort P Thomas C. Effect of different onset thresholds on isometric midthigh pull force-time variables. J Strength Cond Res. (2017) 31:3463–73. 10.1519/JSC.0000000000001765 28002178

[34] Wang R Hoffman JR Tanigawa S Miramonti AA La Monica MB Beyer KS Isometric mid-thigh pull correlates with strength, sprint, and agility performance in collegiate rugby union players. J Strength Cond Res. (2016) 30:3051–6. 10.1519/JSC.0000000000001416 26982977

[35] Jones PA Herrington L Graham-Smith P. Braking characteristics during cutting and pivoting in female soccer players. J Electromyogr Kinesiol. (2016) 30:46–54. 10.1016/j.jelekin.2016.05.006 27295508

[36] Shinchi K Yamashita D Yamagishi T Kazuhiro A Miyamoto N. Relationship between jump height and lower limb joint kinetics and kinematics during countermovement jump in elite male athletes. Sports Biomech. (2024) 23:3454–65. 10.1080/14763141.2024.2351212 38742268

[37] Dos’Santos T Thomas C Jones PA Comfort P. Mechanical determinants of faster change of direction speed performance in male athletes. J Strength Cond Res. (2017) 31:696–705. 10.1519/JSC.0000000000001535 27379954

[38] Hopkins WG Marshall SW Batterham AM Hanin J. Progressive statistics for studies in sports medicine and exercise science. Med Sci Sports Exerc. (2009) 41:3–12. 10.1249/MSS.0b013e31818cb278 19092709

[39] Uysal AA Stone MH Carroll K Faust T. Comparing anthropometric and performance test results across playing levels and evaluating their correlation with game performance in women’s collegiate basketball. J Strength Cond Res. (2025) 39:1177–85. 10.1519/JSC.0000000000005211 40845280

[40] Chen Z Yin M Bishop C Ainsworth B Li Y. Association between lower body qualities and change-of-direction performance: a meta-analysis. Int J Sports Med. (2023) 44:1013–33. 10.1055/a-2117-9490 37364608

[41] Nimphius S Geib G Spiteri T Carlisle D. “Change of direction deficit” measurement in division 1 American football players. J Aust Strength Cond. (2013) 21(Supplement 2):115–7.

[42] Smolarek T Haff GG Poon WCK Nagatani T Barley OR Guppy SN. Dynamic and isometric force-time curve characteristics influencing change of direction performance of state-level netball players. J Strength Cond Res. (2023) 37:2397–404. 10.1519/JSC.0000000000004616 37815246

[43] Alfaro EL Shields JE Zaragoza JA Lopes M Santos D Dawes JJ Correlation between lower-body strength and performance tests among female NCAA division II softball players. Int J Exerc Sci. (2024) 17:212–9. 10.70252/CIQD4720 38665858 PMC11042884

[44] Andersen E Lockie RG Dawes JJ. Relationship of absolute and relative lower-body strength to predictors of athletic performance in collegiate women soccer players. Sports. (2018) 6:106. 10.3390/sports6040106 30274252 PMC6316620

[45] Ahn N Kim H Krzyszkowski J Roche S Kipp K. Influence of the bar position on joint-level biomechanics during isometric pulling exercises. J Strength Cond Res. (2021) 35:1484–90. 10.1519/JSC.0000000000004017 33795603

[46] Dos’Santos T Thomas C Comfort P Jones PA. The effect of angle and velocity on change of direction biomechanics: an angle-velocity trade-off. Sports Med. (2018) 48:2235–53. 10.1007/s40279-018-0968-3 30094799 PMC6132493

[47] Falch HN Rædergård HG Van Den Tillaar R. Relationship of performance measures and muscle activity between a 180° change of direction task and different countermovement jumps. Sports. (2020) 8:47. 10.3390/sports8040047 32290048 PMC7240375

[48] Singh U Leicht AS Connor JD Brice SM Alves A Doma K. Biomechanical determinants of change of direction performance: a systematic review. Sports Med. (2025) 55(9):2207–24. 10.1007/s40279-025-02278-3 40668491 PMC12476310

[49] Leidersdorf E Rauch J Reeves T Borkan L Francis J Storey L Reliability and effectiveness of a lateral countermovement jump for stratifying shuffling performance amongst elite basketball players. Sports. (2022) 10:186. 10.3390/sports10110186 36422955 PMC9697629

[50] Nimphius S McGuigan MR Newton RU. Relationship between strength, power, speed and change of direction performance of female softball players. J Strength Cond Res. (2010) 24:885–95. 10.1519/JSC.0b013e3181d4d41d 20300038

[51] Bissas A Havenetidis K. The use of various strength-power tests as predictors of sprint running performance. J Sports Med Phys Fitness. (2008) 48:49–54. 18212710

[52] Tillin NA Pain MTG Folland J. Explosive force production during isometric squats correlates with athletic performance in rugby union players. J Sports Sci. (2013) 31:66–76. 10.1080/02640414.2012.720704 22938509

